# Implicit hype? Representations of platelet rich plasma in the news media

**DOI:** 10.1371/journal.pone.0182496

**Published:** 2017-08-09

**Authors:** Christen Rachul, John E. J. Rasko, Timothy Caulfield

**Affiliations:** 1 Office of Educational and Faculty Development, Rady Faculty of Health Sciences, University of Manitoba, Winnipeg, Manitoba, Canada; 2 Gene & Stem Cell Therapy Program, Centenary Institute, University of Sydney, Camperdown, Australia; 3 Sydney Medical School, University of Sydney, Camperdown, Australia; 4 Cell and Molecular Therapies, Royal Prince Alfred Hospital, Camperdown, Australia; 5 Health Law Institute, and Faculty of Law and School of Public Health, University of Alberta, Edmonton, Alberta, Canada; University of Pittsburgh, UNITED STATES

## Abstract

Platelet Rich Plasma (PRP) has gained popularity in recent years for treating sports-related injuries and the news media frequently reports on elite athletes’ and celebrities’ use of PRP. We conducted a content analysis of newspaper coverage of PRP in Australia, Canada, Ireland, New Zealand, United Kingdom, and the United States. Findings show that news media coverage of PRP appears most frequently in sports-related stories, and in relation to elite athletes use of PRP. PRP injections are largely portrayed as a routine treatment for sports-related injuries and newspaper articles rarely discuss the limitations or efficacy of PRP. We argue that while news media coverage of PRP exhibits very few common hallmarks of hype, its portrayal as a routine treatment used by elite athletes and celebrities creates an implicit hype. This implicit hype can contribute to public misunderstandings of the efficacy of PRP.

## Introduction

Despite its development and clinical use for over thirty years, platelet rich plasma (PRP) injections have become increasingly popular in the past decade, particularly for treating sports-related musculoskeletal injuries. The injections are widely available at both private and hospital clinics, and as a cosmetic, anti-aging service often referred to as the “vampire facial” [1, 2]. PRP has also garnered the attention of the popular media, particularly its use by elite athletes such as Tiger Woods, members of the Pittsburgh Steelers’ prior to winning the Super Bowl, many professional soccer players [3], and celebrities such as Kim Kardashian [4]. However, despite its popularity, there is little evidence from clinical trials to support this widespread use and, to date, it remains a largely unproven treatment [5–8].

Popular media coverage of new and emerging biotechnologies and therapies has been shown to contribute to the development of “science hype”–that is, the exaggeration of the benefits of science and understatement of any risks or other concerns [9,10]. Recent studies have found that popular media coverage of health and health technologies also favor patient access to health therapies over concerns about efficacy, safety, and even financial limitations [11,12]. In addition to, and perhaps as a consequence of, the popular media contributing to science hype, the media can play a role in shaping public perceptions of and interest in new biomedical technologies [13], and even influence an individual’s health-related behaviors [14]. Media coverage of celebrities’ use of new and emerging biotechnologies and therapies may further heighten the hype surrounding these technologies and therapies [15,16], and also have an impact on health-related decision-making [17–19].

Given the growing popularity of PRP injections and the media coverage of its use by elite athletes and celebrities, we examined the portrayals of PRP in English-language newspapers published in six countries. This analysis seems particularly timely given the growing concerns regarding both how research is presented to the public–especially in the context of regenerative medicine [10]–and the promotion of unproven regenerative therapies [20–23]. How the media represents PRP matters. It may impact public expectations and facilitate the marketing of products and procedures that do not have a strong scientific base.

### Context

At the current time, the efficacy of PRP in the context of most conditions remains unclear, at best. Indeed, in well-controlled clinical trails and meta-analyses where bias was minimized, PRP has been demonstrated to offer no benefit in acute and chronic musculoskeletal injuries [5–8] including: torn rotator cuff [24,25]; chronic Achilles tendinopathy [26,27]; anterior cruciate ligament graft surgery [28]; and acute hamstring muscle injuries [29,30]. While clinical research continues, there are very few conditions for which there is robust evidence to support clinical application. It is also noteworthy that there is no standardized method for production of PRP and many variations exist. This may also account for a lack of consistency in the outcomes of clinical trials.

In the US, for example, PRP is an autologous human blood product that is regulated in the USA by the Center for Biologics Evaluation and Research (CBER) within the Food and Drug Administration. Currently PRP is approved by CBER to assist surgical procedures involving bone grafts. All other medical procedures involving PRP are regarded as off-label and typically will not be reimbursed by insurers [31].

## Methods

To examine popular media portrayals of PRP, we conducted a qualitative content analysis of English-language newspaper articles published in six countries. We searched for newspaper articles published in the top 3 newspapers by circulation in Canada, USA, UK, Ireland, Australia, and New Zealand based on access to full coverage on the Factiva database (S1 File). We searched for articles published between the January 1, 2009 and December 31, 2015 using the terms “platelet rich plasma” and “PRP”. This search yielded 464 newspaper articles. Irrelevant articles were then excluded from the data set. Articles were deemed irrelevant or excluded if they were stock market updates, referred to the use of PRP injections in animals, or included the acronym PRP that did not stand for platelet rich plasma. The resulting data set included 307 relevant articles.

We developed an inductive coding frame using methods from previous studies conducted by our team [11,12]. The coding frame was designed to elicit information about the main frame of articles, the person or population on which the articles focus, descriptions of PRP, and the portrayal of the effectiveness, benefits, and limitations of PRP, including any evidence cited for its effectiveness or lack thereof.

One team member coded all 307 articles and a second coder who was previously uninvolved in the project coded 30 randomly selected articles (~10%) to determine reliability of coding. Inter-coder agreement was calculated using methods from Miles and Huberman [32] that calculates agreement as total agreements/(total agreements + disagreements). Agreement was between 83.3% and 100.0% for all coding frame items, with average agreement at 91.9%.

## Results

News articles that mention PRP were published from 22 to 74 times per year. There was a spike in 2010 (53 articles) when Dr. Anthony Galea’s drug doping scandal made the news and his treatment of Tiger Woods, which included PRP, came under scrutiny. There is another spike in 2013 (61 articles) and 2014 (74 articles) when “blood-spinning” for athletes and “vampire facials” for celebrities such as Kim Kardashian received considerable media coverage.

News coverage of PRP was significantly different between the countries, *X*^2^(5, *N* = 307) = 187.83, *p* = < 0.01, with US newspapers providing more coverage than the other countries. Canada had the second most coverage, but this appears to be largely because Dr. Anthony Galea is Canadian. The majority of the news articles (70%) were published in the sports sections of the newspapers. Coverage in other newspaper sections was less than 10% for each of the different sections in which articles appeared, for example, 25 articles (8.1%) appeared in news sections and only 12 articles appeared in health or science sections (3.9%).

### Focus of news articles

**Fig 1:**
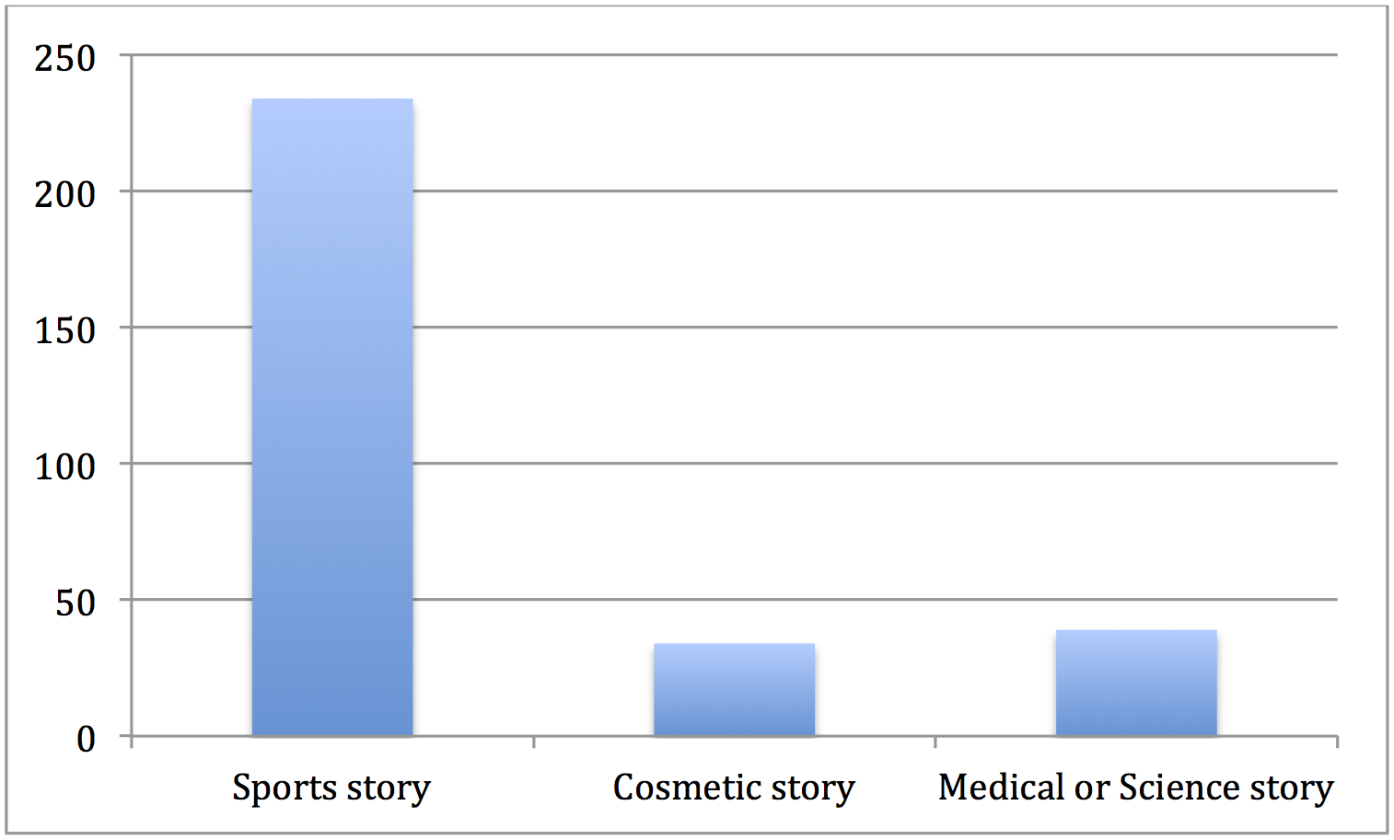
# of articles by main frame.

Articles were coded according to how they framed stories that mention PRP. Over 75% of articles were sports-related stories (234 articles, 76.4%), 12.7% of articles (39) were medical or science-related stories, and 11.1% of articles (34) were cosmetic-related stories. There are significant differences in the main frames of articles published in each country, *X*^2^(10, *N* = 307) = 118.98, *p* = < 0.01. A large majority of articles from Australia (97.1% of all Australian articles), USA (87.1% of all US articles), and Canada (79.4% of all Canadian articles) were sports-related articles (Fig 1). In contrast, 81.8% of articles from New Zealand were cosmetic stories and less than 10% were sports-related.

In addition, articles were coded for the person or population on whom the articles were focused. Almost 70% of the articles focused on athletes or mentioned athletes’ use of PRP (Table 1). Another 11.1% of articles focused on a doctor who provides PRP injections, but 31 of these 34 articles focused on Dr. Anthony Galea who was involved in a doping scandal in 2010. Finally, 17.9% of articles did not focus on a specific population or person.

**Table 1:** Population or person that articles focus on.

Population or Person	#	%
Athletes	209	68.1%
No specific person or population	55	17.9%
Doctor providing treatment	34	11.1%
Patients	5	1.6%
Celebrities	4	1.3%

### Portrayal of PRP

Articles were coded for descriptions of PRP, the conditions that PRP injections treat, and whether PRP is portrayed as a routine, new or cutting-edge, or experimental treatment. Descriptions of PRP that ranged from a simple description of the use of one’s own blood to more informative descriptions about platelets and growth hormones were included in 119 articles (38.8%). In addition, 199 articles (64.8%) included a description of the procedure for PRP injections that ranged from basic descriptions about injections (99 articles, 32.2%) to more detailed descriptions (100 articles, 32.5%).

Coding of articles elicited 20 different conditions or ways of describing what PRP injections were used to treat. Over 30% of articles never specified the condition that PRP treats or was used to treat or they included multiple conditions, and 13.6% of article referred to injury (usually sports-related), but did not specify what kind of injury PRP treats or treated. The most common condition that was specified was tears (17.5%), and these generally referred to muscle or tendon tears.

**Fig 2:**
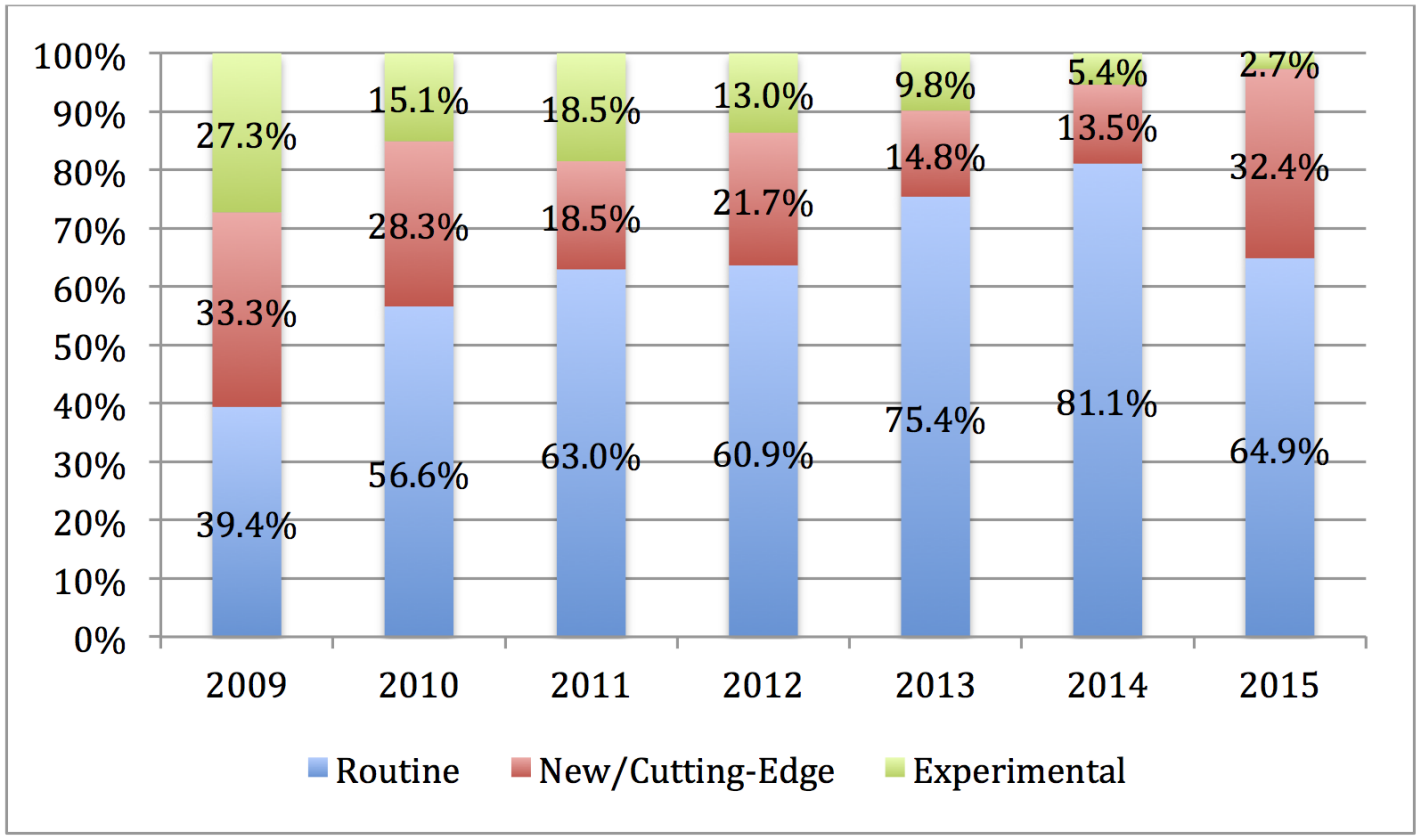
Portrayal of PRP over time as routine, new, or experimental.

**Fig 3:**
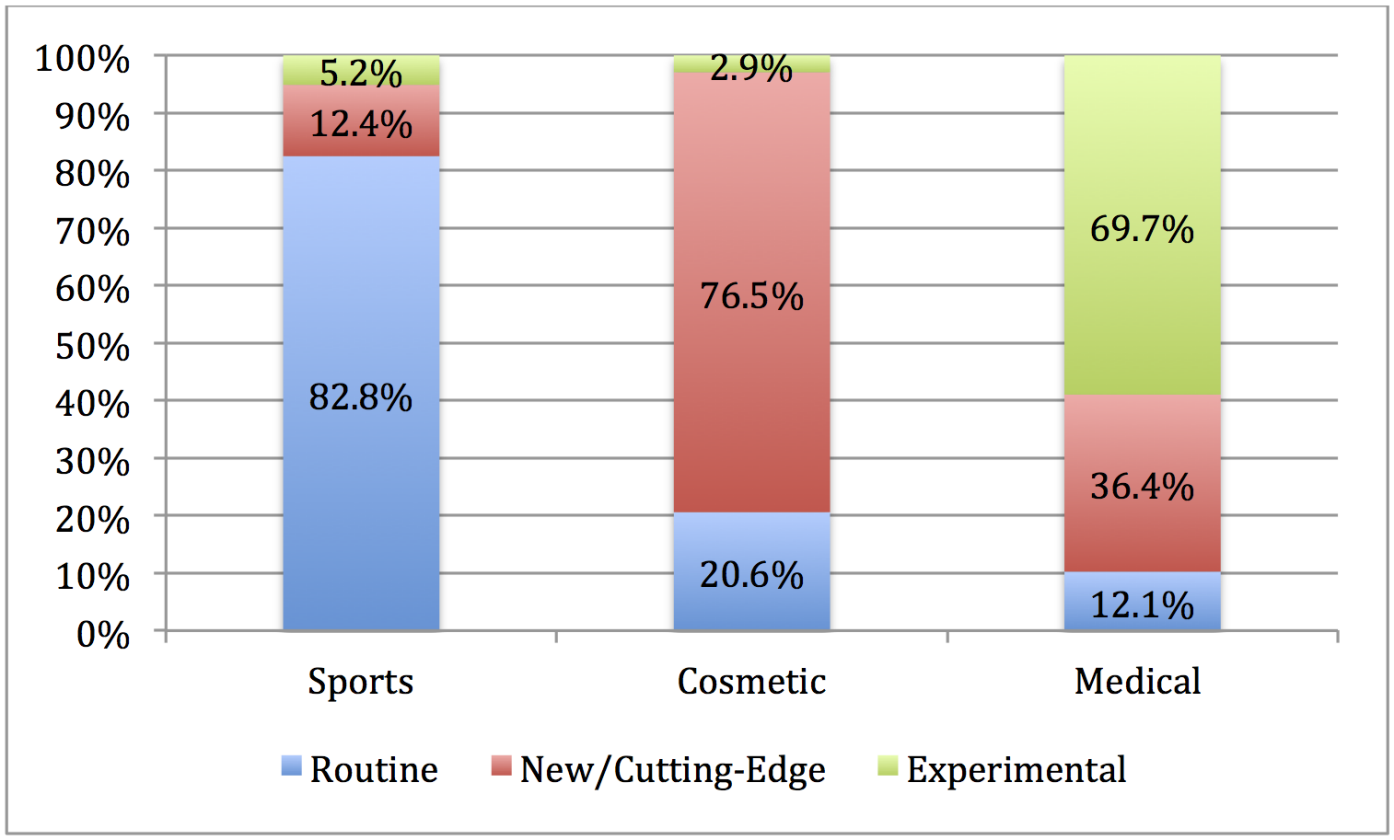
Portrayal of PRP as routine, new, or experimental by main frame of story.

In addition, PRP injections were most often portrayed as a routine procedure (204 articles, 66.4%), but 67 articles (21.8%) portrayed PRP as new or cutting edge and 36 articles (11.7%) portrayed it as experimental. However, these portrayals changed over time, *X*^2^(12, *N* = 307) = 30.15, *p* = 0.03, where PRP was increasingly portrayed as a routine treatment over time (Fig 2). There were also significant differences between the main frame of a story and the portrayal of PRP as routine, new, or experimental, *X*^2^(4, *N* = 307) = 181.00, *p* = < 0.01 (Fig 3). The majority of articles (193, 82.5%) whose main frame was sports-related portrayed PRP as a routine treatment. In comparison, 76.5% of articles (26 articles) whose main frame was cosmetic portrayed PRP injections as new or cutting-edge. Over half of the articles with a medical/scientific frame portrayed PRP as experimental (23 articles, 59.0%).

We also made note of key descriptive words and phrases that were used to describe PRP that might also indicate or create hype around the treatment. For example, we noted descriptive words and phrases intended to generate excitement such as “cutting edge” or “breakthrough,” and words or phrases that might relate PRP to readers’ values and interests such as “natural.” Only 66 articles (21.4%) used adjectives or descriptive phrases to describe PRP, and generally only a handful of articles used similar phrases. For example, 10 articles (3.2%) used the word “natural” to describe PRP, 12 articles (3.9%) described it as “cutting-edge” or “innovative”, and about 5 articles (1.6%) described it as “controversial”.

### Effectiveness of PRP

Articles were coded for whether they portrayed PRP as effective, ineffective, or whether its effectiveness is unclear. PRP was portrayed as effective in 23.8% of articles (73), 22.8% of articles (70) mentioned that the effectiveness of PRP is unclear or uncertain, and 6.5% of articles (20) mentioned that it was ineffective. Almost half of the articles (144, 46.9%) made no explicit mention about whether or not it was effective.

Articles were also coded for whether any evidence was presented regarding the effectiveness of PRP. Only 36.8% (113) of articles cited any evidence for claims about the effectiveness of PRP therapy. Most of these articles (72, 23.5% of all articles) provided anecdotal evidence and 36 articles (11.7%) cited evidence from a clinical trial or medical study.

### Benefits and limitations of PRP

Just over half of the articles (171 articles, 55.7%) explicitly mentioned any benefits. The most common benefit of PRP that was mentioned in articles was that it accelerates healing (61 articles, 19.9%) or that it promotes healing (58 articles, 18.9%). Just over a quarter of the articles mentioned any risks or limitations associated with PRP (85 articles, 27.7%). The most common limitations of PRP mentioned in articles were the lack of evidence supporting the efficacy of PRP (34 articles, 11.1%) and 12 articles (3.9%) also indicated that more research on PRP was needed. Interestingly the second most common limitation was that it is associated with or implicated in doping scandals (26 articles, 8.5%).

## Discussion

The qualitative content analysis of newspaper articles revealed that popular media coverage of PRP injections is largely framed as a sports-related story and is also commonly portrayed as a routine treatment. Not surprisingly, articles that portrayed PRP as routine commonly appeared in sports-related stories, which is in contrast to cosmetic-related stories that tended to portray it as new and cutting-edge and medical or science stories that portrayed it as experimental. Interestingly, less than a quarter of the articles mentioned that the PRP injections were effective while almost half of the articles made no mention of effectiveness in either direction.

There is a seeming contradiction between the common portrayal of PRP as a routine treatment and a lack of discussion of its effectiveness in newspaper articles. This contradiction raises interesting questions about the role of popular media in creating and perpetuating hype around a treatment that has, despite decades of clinical use, very little evidence for its efficacy outside of bone grafting. Hype in popular media has often been associated with phrases such as “cutting-edge” and “breakthrough” [33], and with stories that highlight the benefits while omitting the risks of new or emerging biotechnologies. However, the media coverage of PRP displays relatively few of these hallmarks of hype.

Despite few hallmarks of hype, the frequent association of the use of PRP with elite athletes and celebrities can result in its own kind of hype [34]. Here we argue that framing PRP as a routine treatment with very little critical discussion or explicit hype, in combination with frequent reporting of the use of PRP by elite athletes and celebrities creates an *implicit hype*. That is, hype around PRP has developed not by framing it as a breakthrough or through other common features of hype, but through a lack of critical discussions about evidence for efficacy in stories about its routine use by celebrities. For the public, the take away message from these stories may be that celebrity athletes use PRP because it works–and because the stories are very often in the sports portions of the paper, there is very little critical analysis of the relevant science. This implicit hype in popular media arguably has the same impact on public perceptions of new or unproven therapies as more explicit hype, and may influence individuals to choose costly and largely unproven.

Our analysis also raises the question as to why there have been so few properly controlled randomized clinical trials with sufficient power to reach convincing statistical conclusions despite decades since PRP was first used. One possible inference is that implicit hype maintains a clinical demand that discourages those with vested interests to seek a definitive clinical conclusion.

To our knowledge, this is the first analysis of how PRP is represented in the popular press. This study highlights some interesting and worrisome trends, including the framing of PRP as routine and the possible existence of implicit hype. However, there are limitations to our approach that should be considered, including the narrow scope of our analysis. We did not, for example, explore how PRP is represented on social media or on clinic websites and we conducted an analysis within a limited time frame for a field that is continually growing and changing. In addition, due to limitations of the Factiva database we were unable to assess any third party influence or financing of news articles. Future research could focus on the possible impact of implicit hype, such as exploring public reaction to stories involving the use of therapies by high profile athletes and celebrities.

There is growing concern about public misrepresentations regarding the clinical readiness of emerging technologies [10,35]. It has also been noted that media reports of their use by high profile athletes may contribute to public confusion regarding efficacy, such as in the area of stem cells [15,36]. This study suggests PRP is also being represented in a less than ideal fashion and in a manner that may contribute to public misunderstanding about the efficacy of this technology. The implicit hype generated by news media representations of PRP may also lead to other unintended and negative consequences for policy and practice as it continues to move through the notorious “hype pipeline” [9].

## Supporting information

S1 File Supplementary methods. (PDF)
