# Supplementary material for: Implicit hype? Representations of platelet rich plasma in the news media
Source: PLoS One. 2017 Aug 9;12(8):e0182496. doi: 10.1371/journal.pone.0182496 (PMC5549909; doi:10.1371/journal.pone.0182496)
Supplement: S1 File — (PDF) [file pone.0182496.s001.pdf]

S1. Supplementary Methods

Data Set Characteristics:

Newspaper	# of articles
**Australia**	
Daily Telegraph (Aus)	9
The Australian	14
Herald Sun	11
**Canada**	
Globe and Mail	21
National Post	24
Toronto Star	23
**Ireland**	
Irish Independent	11
Irish Examiner	3
Irish Times	11
**New Zealand**	
New Zealand Herald	7
Dominion Post	2
The Press	2
**United Kingdom**	
Daily Telegraph (UK)	8
Daily Mail	19
The Times	10
**United States**	
Wall Street Journal	43
USA Today	23
New York Times	66

Circulation numbers were retrieved from the following:

Australia:
http://www.roymorgan.com/industries/media/readership/newspaper-readership
http://www.onlinenewspapers.com/Top50/Top50-CurrentAustralia.htm

Canada:
http://newspaperscanada.ca/about-newspapers/circulation/daily-newspapers/

Ireland:
http://newsbrandsireland.ie/data-centre/circulation/

New Zealand:
http://newspaper.abc.org.nz/audit.html?org=npa&publicationid=%25&mode=embargo&npa_admin=1&publicationtype=19&memberid=%25&type=%25

UK:
http://www.pressgazette.co.uk/national-newspaper-circulations-may-2015-mail-sunday-overtakes-sun-sunday-times-remains-biggest-growing/

USA:
http://auditedmedia.com/news/research-and-data/top-25-us-newspapers-for-march-2013/
http://www.journalism.org/media-indicators/newspapers-top-25-u-s-daily-newspapers-with-digital-editions/
